# Clinical and genetic aspects of defects in the mitochondrial iron–sulfur cluster synthesis pathway

**DOI:** 10.1007/s00775-018-1550-z

**Published:** 2018-04-05

**Authors:** A. V. Vanlander, R. Van Coster

**Affiliations:** Division of Pediatric Neurology and Metabolism, Department of Pediatrics, Ghent University Hospital, Prinses Elisabeth ziekenhuis, 3K12D, secretariaat kinderneurologie, Corneel Heymanslaan 10, Ghent, 9000 Belgium

**Keywords:** Iron–sulfur clusters, Mitochondria, Phenotype, OXPHOS

## Abstract

Iron–sulfur clusters are evolutionarily conserved biological structures which play an important role as cofactor for multiple enzymes in eukaryotic cells. The biosynthesis pathways of the iron–sulfur clusters are located in the mitochondria and in the cytosol. The mitochondrial iron–sulfur cluster biosynthesis pathway (ISC) can be divided into at least twenty enzymatic steps. Since the description of frataxin deficiency as the cause of Friedreich’s ataxia, multiple other deficiencies in ISC biosynthesis pathway have been reported. In this paper, an overview is given of the clinical, biochemical and genetic aspects reported in humans affected by a defect in iron–sulfur cluster biosynthesis.

## Introduction

In eukaryotes, the Fe/S-cluster biosynthesis machinery is classically divided into the mitochondrial iron–sulfur cluster assembly (ISC) and export machinery, and cytosolic iron–sulfur protein assembly (CIA) system. Nowadays, 9 CIA proteins and 20 ISC proteins are known to assist the major steps in biogenesis. All steps in these machineries are evolutionarily conserved from yeast to man. Once incorporated into their target protein, Fe/S-cluster function as catalysts or take part in electron transfer. They also serve as sulfur donors in lipoate and biotin cofactor biosynthesis [1]. Fe/S-cluster-bearing proteins are located in the mitochondria and in the cell nucleus, where they play a role in gene expression regulation [2]. Moreover, proteins involved in DNA replication, DNA repair (Pol α, Pol ε, Pol δ, and Pol γ) and those functioning as DNA helicase harbor Fe/S clusters [3]. The cytosolic ABC protein ABCE1 required for ribosome assembly and protein translation has two [4Fe/4S] clusters [4]. Apart from being synthesized and incorporated as cofactor into apoproteins, iron–sulfur clusters also serve as redox center, as they are incorporated in the complexes I, II, and III of the oxidative phosphorylation system (OXPHOS), embedded in the inner mitochondrial membrane.

Considering the pleiotropic subcellular localization and the essential role of the Fe–S-cluster-bearing enzymes in cell viability, it is easy to understand that faulty synthesis and lack of insertion of these inorganic elements can have detrimental effects on human health. An overview will be given of the genetic and clinical aspects of the molecular defects located in the biosynthesis pathway of iron–sulfur clusters in the mitochondria. Until now, diseases resulting from pathogenic mutations in proteins involved in CIA have not been reported.

## Mitochondrial iron–sulfur biosynthesis (ISC)

**Fig. 1:**
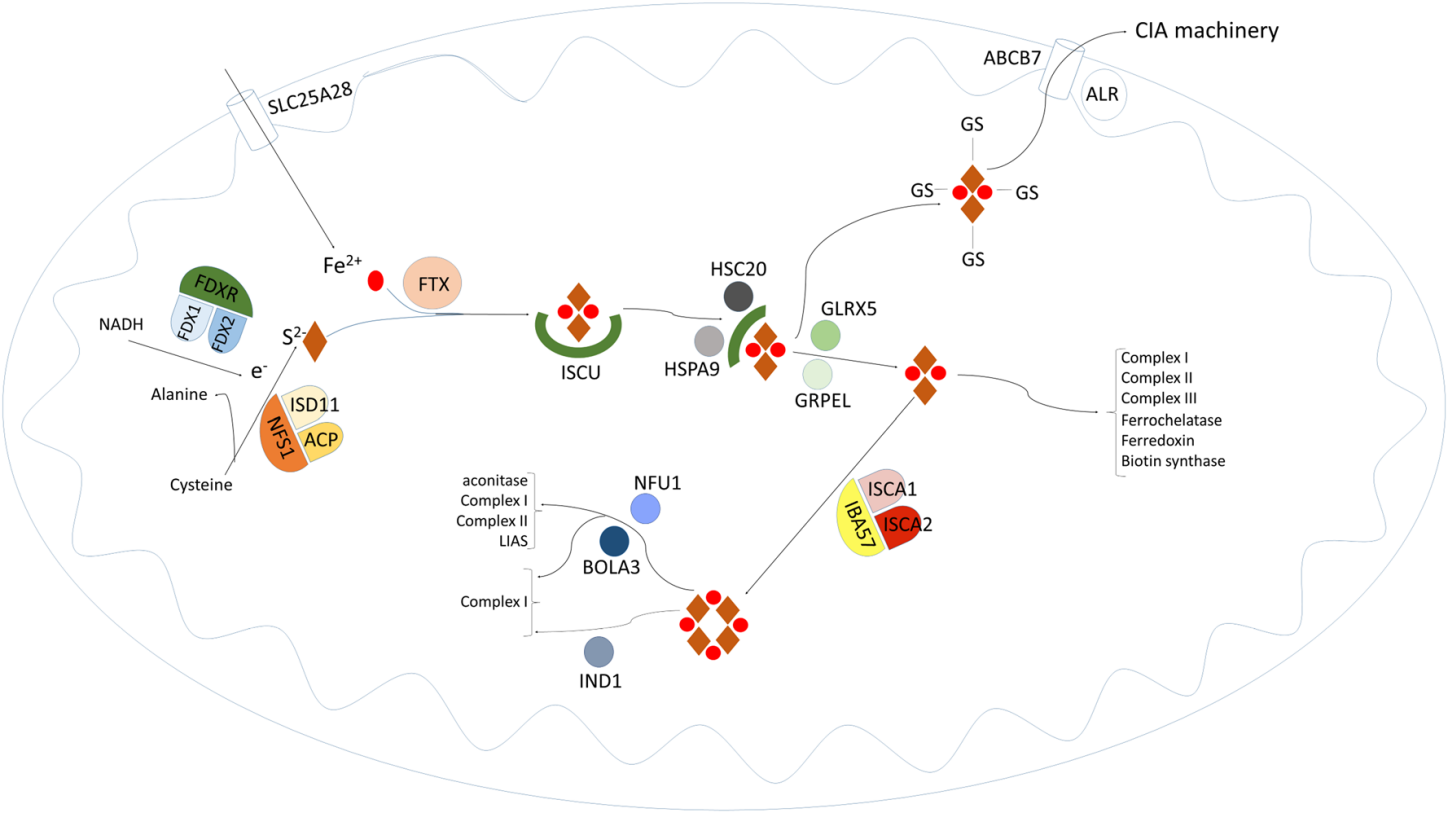
Schematic representation of the intramitochondrial iron–sulfur cluster biosynthesis pathway (see text for details)

ISC can be divided into three major steps. The first part encompasses formation of a [2Fe–2S] Fe/S cluster on a scaffold protein. Subsequently, the cluster released from the scaffold protein by dedicated chaperones is maintained in a gluthatione (GSH)-dependent fashion. The synthesized product is further processed intramitochondrially or exported into the cytosol to be processed by CIA. The exact nature of the exported structure is unknown, but it might be a glutathione-stabilized [2Fe–2S] ([2Fe–2S](GS)_4_^2−^) [5]. The export of this structure is mediated by ABCB7 in cooperation with ALR, an FAD-dependent sulfhydryl oxidase. The implication of ALR is, however, still a matter of debate, as a study in yeast could not show impaired cytosolic iron–sulfur cluster assembly [6]. The mitochondrial machinery can synthesize [2Fe–2S] or [4Fe–4S] clusters and incorporate these into the appropriate apoproteins (Fig. 1). For a detailed description of the iron–sulfur cluster pathway and the specific role of each enzyme within this pathway, we refer to other papers within this issue.

Faulty ISC synthesis can lead to mitochondrial failure, preferentially affecting high energy consuming organs, i.e., central nervous system, skeletal muscle, heart muscle, and liver. A deficiency in ISC synthesis can thus mimic clinical phenotypes of oxidative phosphorylation defects (Table 1). Considering the intricate relationship between ISC biosynthesis and cellular iron homeostasis, ISC deficiencies can lead to iron accumulation.

**Table 1:** Overview of the reported ISC related diseases showing the causative genes, the clinical, biochemical and morphological features

Gene	Clinical features	Biochemical and morphological features	References
*SLC25A37*:	RARS (myelodysplastic syndrome with isolated anemia ineffective erythropoiesis)	Increased expression of mitoferrin 1	16
*ISCU*:	Myopathy with exercise intolerance Severe myopathy with hypertrophic cardiomyopathy	Decreased complex I, II, and III activity (muscle) Normal lipoylation Increased lactate	[13–15]
*FXN*:	Friedreich’s ataxia (progressive ataxia, dysarthria, diabetes mellitus, cardiomyopathy) < 20 years	Aconitase deficiency Decreased complex I, II, and III activity (heart) Mitochondrial iron overload	[21, 24]
*NFS1*:	Cardiomyopathy, epilepsy Hypotonia, multiple organ failure, epilepsy Developmental delay, hypotonia	Decreased complex II and III activity (muscle, liver) Absence of LYRM4	30
*LYRM4*:	(Transient) hypotonia ± epilepsy	Decreased complex I, II, and III activity (muscle, liver) Decreased expression: aconitase, ferrochelatase	31
*FDXR*:	Auditory neuropathy and optic atrophy	Decreased complex I, III, (IV, V) (fibroblasts) Iron accumulation, aconitase deficiency (fibroblasts)	33
*FDXL2*:	Myopathy (cramps, rhabdomyolysis, myoglobinuria)	Increased lactate Decreased complex I, II, and III activity (muscle, liver) Aconitase deficiency	34
*HSPA9*:	EVEN-PLUS syndrome	Ineffective hematopoiesis (zebrafish)	[38, 39]
*GLRX5*:	Lower limb spasticity, optic nerve atrophy Congenital sideroblastic anemia ± diabetes mellitus type1 ± cirrhosis ± hypogonadism	Increased lactate (serum) and glycine (CSF) Normal OXPHOS activity (muscle) Defective lipoylation (fibroblasts) Ring sideroblasts; iron accumulation (fibroblasts) Increased ferritin (serum)	[7, 41–43]
*ABCB7*:	Sideroblastic anemia, ataxia ± cerebellar atrophy Female carriers: anemia	Mild hypochromic, microcytic anemia Variable ferritin (serum) Normal OXPHOS activity (fibroblasts)	[45, 46, 48]
*ALR*:	Developmental delay, hypotonia (± dystonia, choreic movements), congenital cataract ± hearing loss	Increased lactate (serum) Decreased complex I, II, III, and IV activity (muscle, fibroblast)	[51–53]
*ISCA1*:	Regression, seizures, progressive leukodystrophy	Increased lactate	57
*ISCA2*:	Diffuse progressive leukodystrophy	Isolated complex I deficiency (fibroblasts) Decreased complex I, II, and III activity (HeLa) Decreased aconitase activity (HeLa)	58
*IBA57*:	Microcephaly, hypotonia, encephalopathy, dysmorphism Regression, leukodystrophy Spastic paraparesis, peripheral neuropathy ± optic nerve atrophy (SPOAN)	Increased lactate and glycine (serum, CSF) Faulty lipoylation Decreased complex I, II, and IV activity (muscle, fibroblasts) Decreased aconitase activity (fibroblasts) Decreased complex I, II, and III activity (HeLa)	[60–64]
*NFU1*:	Failure to thrive, pulmonary hypertension, hypotonia, leukodystrophy Pulmonary hypertension, regression Bilateral optic atrophy Epilepsy	Increased glycine (serum and CSF) Increased lactate (serum) Faulty lipoylation Decreased complex II and PDHC activity (muscle) (variable OXPHOS deficiencies in tissues)	[68, 70–72]
*BOLA3*:	Epileptic encephalopathy, cardiomyopathy Hypertrophic cardiomyopathy, regression, epilepsy Spasticity, ataxia, regression, epilepsy	Increased glycine (serum, CSF) Increased lactate (serum) Faulty lipoylation Decreased complex I and II (fibroblasts, muscle) Normal PDHC and aconitase activity	[41, 66, 73]
*NUBPL*:	Encephalopathy Ataxia, spasticity Developmental delay, ataxia, spasticity, white matter alterations	Increased lactate (serum, CSF) Deficient complex I activity (muscle, fibroblasts	[74, 76]

### De novo synthesis of [2Fe–2S] clusters

One of the mitochondrial matrix proteins dedicated to Fe–S-cluster synthesis is ISCU [7]. The ferrous iron required for Fe/S synthesis is imported into the mitochondria through the mitochondrial solute carriers mitoferrin 1 (SLC25A37) and 2 (SLC25A28). In contrast to mitoferrin 1, which is exclusively expressed in developing erythroid cells, mitoferrin 2 is widely expressed [8]. How iron gets into the scaffold protein is not totally elucidated yet, but frataxin (FXN) plays an important role in this process at least in humans. Interaction of frataxin with ISCU is important for ISC biosynthesis and seems to be iron dependent [9]. The sulfur component is delivered through desulfuration of cysteine into alanine by cysteine desulfurase (NFS1), acting in a dimer conformation, in association with the cofactor pyridoxal 5’ phosphate. In addition, NFS1 requires association with a heterodimer composed of ISD11 (encoded by *LYRM4*) and acyl carrier protein (ACP) [10] for stabilization [11].

#### ISCU

As ISCU functions as a scaffold protein at the start of Fe/S-cluster synthesis, defective ISCU is predicted to impair overall cluster synthesis resulting in large downstream effects, often with lethal consequences. This was confirmed in mice [12]. However, tissue specific splicing and partial enzymatic impairment are found in subjects harboring pathogenic variants in *ISCU*. ISCU deficiency can cause myopathy with exercise intolerance and lactic acidosis, which is called the Swedish type myopathy. Subjects may experience cramps and show rhabdomyolysis. Interestingly, most of the affected subjects carry the common homozygous intronic mutation (7044G > C, or IVS5 + 382G-C), and all of them (except for one Norwegian) are native from a region in Northern Sweden, explaining the denomination of Swedish myopathy [13]. In 2009, two siblings were identified with an exonic missense mutation (149G > A, Gly50Glu) in a compound heterozygous state, which includes the common intronic mutation. They had a more severe phenotype with early onset (around the age of 2 years) of severe muscle weakness and muscle wasting, and hypertrophic cardiomyopathy [14]. Very recently a dominant mode of inheritance (c.287G > T, pGly96Val) was reported in an Italian subject presenting with ptosis, hypotonia and exercise intolerance, showing worsening over time [15]. Biochemical features were different from previously reported cases, including complex IV deficiency in addition to the classically reported complex I, II, and III deficiencies [15]. Analysis of transcript expression showed that the highest level of mutant transcript was in skeletal muscle (80%), while liver and heart had lower levels (46 and 30%, respectively), explaining the tissue specific phenotype of this disease [12].

#### SLC25A37 and SLC25A28

Disease-causing mutations have not been detected yet in genes encoding these proteins. However, in the subjects with refractory anemia and ring sideroblasts (RARS), increased expression of mitoferrin 1 (SLC25A37) in bone-marrow mononuclear cells was found [16]. RARS is a form of myelodysplastic syndrome leading to isolated anemia, hypochromic erythrocytes, hyperplastic ineffective erythropoiesis and mitochondrial ferritin accumulation in erythroid precursor cells.

#### FXN

Being involved early in the ISC synthesis pathway, a decrease of frataxin protein has an impact on the overall Fe/S-cluster synthesis. Indeed, frataxin null mutations were shown to be lethal in mice [17]. Defective frataxin protein is seen in Friedreich’s ataxia. The latter is caused by the presence of a triplet repeat expansion (GAA) in the first intron of the *FXN* gene in the homozygous or in a compound heterozygous state with a missense or nonsense mutation [18, 19]. The disease is characterized by progressive ataxia, the absence of lower limb tendon reflexes, dysarthria, limb weakness leading to loss of ambulation after several years, decreased vibration sense, scoliosis, diabetes mellitus and cardiomyopathy. The neurological symptoms reflect specific vulnerability of dorsal root ganglia, sensory peripheral nerves, corticospinal tract and dentate nucleus [20]. The age of onset is before 20 years. The length of the triplet expansion correlates directly with left ventricular wall thickness [21] and inversely correlates with age of onset and faster exacerbation of symptoms [22]. The affected subjects become ultimately wheelchair bound and cardiomyopathy is often the cause of fatal outcome. Cardiomyopathy seldom causes death before neurological symptoms are fully developed [23]. In accordance with the early involvement of the frataxin protein in ISC biosynthesis pathway, deficiencies of aconitase and of the OXPHOS complexes I, II, and III have been reported in subject’s cardiomyocytes [24]. Mitochondrial iron accumulation was another striking finding. In cultured skin fibroblasts from of Friedreich’s ataxia patients, the activities of complexes I and II were decreased [25].

Importantly, Friedreich’s ataxia is the first iron–sulfur cluster deficiency for which therapeutic options are being developed. Currently, 51 clinical trials are going on or have recently been completed. These are studying different therapeutic approaches aiming (a) to reduce intramitochondrial oxidative stress (idebenone, coenzymeQ, vitamin E, iron chelators), (b) to enhance frataxin endogenous expression (erythropoietin, pioglitazone), or (c) to increase FRDA gene expression (HDAC inhibitors, interferon γ). For further detailed information on this topic, we refer to recently published papers [26, 27]. Some of the proposed strategies, alone or combined, showed improvement on disease rating scales, but are not disease-modifying or curing. However, more promising results are emerging from gene therapy. In a conditional mouse model with complete *Fxn* deletion in cardiac muscle, intravenous administration of adeno-associated virus (AAV) rh10 vector expressing human FXN intravenously prevented occurrence of cardiomyopathy or completely restored heart function [28]. Increased frataxin expression in patient derived lymphoblast was observed after excising the GAA expansion repeat in one allele using zinc finger nuclease [29].

#### NFS1

Not much is known about the clinical characteristics of an NFS1 protein defect in humans as only one report was published until now describing three subjects from consanguineous descent all sharing the same homozygous missense mutation, c.215G > A, p.Arg72Gln [30]. This conserved residue was recognized to be an important residue for the hydrogen bond formation between NFS1 and ISD11 [10, 11]. The first subject presented at 7 months of age with lethargy, myocardial failure, and generalized seizures during an infectious episode ultimately leading to fatal outcome 3 days later. The second subject presented with hypotonia and feeding problems, and developed multiple organ failure, as well as focal seizures due to cerebral infarction. Heart failure was the cause of death at the age of 7 months. The third subject who was started on vitamin supplementation since the age of 6 months was still alive at 11 years and suffered from mild developmental delay and truncal and limb hypotonia [30]. Biochemical features included increased lactate in body fluids and decreased complex II and III activity in skeletal muscle and liver (complex I not tested individually) [30].

#### ISD11

LYRM4 encodes iron–sulfur protein biogenesis desulfurase interacting protein 11kDa (ISD11). Until now, only two subjects were reported with homozygous pathogenic missense variant in *LYRM4*. Although both harbored the same genotype, their phenotypes were different. One subject was alive at 20 years of age without symptoms, while the other died at the age of 2 months. The first presented with respiratory distress and hypotonia during the neonatal period. Thereafter, he improved gradually and was lost to follow-up, but reevaluation at age 20 years showed no remarkable clinical anomalies. The latter suffered from neonatal respiratory distress and had hepatomegaly. She developed seizures. Her clinical condition deteriorated and ultimately became fatal. Both subjects showed no anomalies on cerebral imaging [31]. The different outcomes in both individuals could possibly be explained by the availability of sulfur sources during the first weeks of life. Indeed, availability of cysteine, the major sulfur source, is restricted in the neonatal period due to reduced activity of hepatic cystathionase [32]. Affected individuals showed increased lactate in body fluids due to impaired complex functioning of the OXPHOS complexes I, II, and III in skeletal muscle and liver. In both tissues, cytosolic and mitochondrial aconitase and also ferrochelatase showed decreased expression. Incorporation of the mutation in *S. cerevisiae* resulted in growth restriction. In *E. coli*, the variant had no impact on the oligomerization of ISD11 with its partner protein NFS1. The enzymatic desulfurase activity was severely impaired in the same model [31].

#### FDXR

FDXR deficiency has only recently been reported. In one paper, eight individuals from four different families were described. The core clinical features were restricted to sensorineural hearing loss (auditory neuropathy) and optic atrophy. Age of onset for hearing impairment ranged from five to 20 years and from 2 to 36 years for visual impairment. Brain imaging revealed no abnormalities. All individuals had missense mutations, except for one who had a nonsense mutation in compound heterozygous status. Biochemical analysis in affected subjects was performed in cultured skin fibroblasts showing impaired complexes I, III, IV, and V in one subject and impairment of complexes I and III in the other. The absence of complex II involvement was remarkable, certainly considering the decreased expression of SDHB. Finally, iron overload, in combination with decreased IRP1 content, was noticed [33].

#### FDX2

Only one subject with a defect in FDX2 (encoded by the *FDXL*) was reported, so far. A homozygous missense mutation in the start codon resulted in a severe decrease of expression of *FDXL*. The subject suffered from myopathy characterized by episodes of acute cramps, rhabdomyolysis, and myoglobinuria after moderate physical activity. During follow-up, a slowly progressive muscle weakness was noticed. The mental capacities were not altered. During acute episodes, serum lactate was increased. OXPHOS activity analysis in skeletal muscle showed typical features of Fe/S-cluster deficiency with decreased activities of complexes I, II, and III, and a decreased activity of the Fe/S-cluster matrix enzyme aconitase [34].

Interestingly, the clinical presentation of FDX2 deficiency mimics the phenotype of individuals with ISCU deficiency. Tissue specific splicing cannot be an explanation for the skeletal muscle specific phenotype as FDX2 is expressed ubiquitously. The authors suggest that FDX2 is not a vital component in Fe–S biogenesis and that FDX1 may partly take over the function in basal conditions, but not in extreme conditions [34].

### [2Fe–2S] cluster release

Cluster release starts by binding of the J-type co-chaperone HSCB (HSC20, Jac1), and HSPA9 (mortalin, HSPA9B) to the ISCU-Fe/S, resulting in loosening of the [2Fe–2S] cluster in an ATP dependent process [36]. In addition, GLRX5, possessing a [2Fe–2S] cluster itself, binds to the complex in a dimeric conformation and recruits GRPEL1 for final cluster release [35, 36]. Synthesized [2Fe–2S] clusters or intermediate elements meant for further processing by the cytosolic iron–sulfur machinery are exported by the ABCB7 translocator. The FAD-dependent sulfhydryl oxidase ALR is thought to support the export [37]. It is generally accepted that proper cytosolic ISC synthesis relies on exported mitochondrial intermediates. Subsequently, we can conclude that all protein deficiencies occurring before this stage can lead eventually to disruption of cytosolic ISC synthesis, and ultimately to mitochondrial iron overload.

#### HSPA9

This heat shock protein is associated with cluster release. It is supposed to have many other functions, including correct folding of proteins after being imported into the mitochondria. Three subjects with a HSPA9 deficiency carried a homozygous missense mutation or missense mutation in a compound state with a nonsense mutation. Affected subjects presented overlapping symptoms recollected in the acronym EVEN-PLUS syndrome, reflecting epiphyseal, vertebral, ear and nose malformation, plus associated findings [38]. Indeed, all subjects presented ‘bifid’ distal femurs and epiphyseal dysplasia of the femur head, resulting in short stature, bilateral microtia and hypoplastic nasal bones. One subject showed vertebral coronal clefts and another lateral vertebral clefts. Other findings comprised arched eyebrows with mild synophrys and atrial septum defects for all reported patients. Two of them had anal atresia and a small area of aplasia cutis. One had hypodontia, which is another feature of ectodermal tissue involvement. Only one subject presented with developmental delay and had abnormal cerebral imaging (dysgenesis of the corpus callosum). This subject also had vesico-ureteral reflux and kidney nephropathy [38]. Biochemical features in affected subjects were not provided, except for the reported anemia. Indeed, a HSPA9 deficient zebrafish, called ‘crimsonless’, showed ineffective hematopoiesis and also deleterious effects on early development of musculature, fins and internal organs leading to death at the 72 hpf stage [39]. An acquired interstitial deletion of the long arm of chromosome 5 [del(5q)] creating haploinsufficiency for a large set of genes including HSPA9 haploinsufficiency is a known cause of myelodysplastic syndrome characterized by ineffective hematopoiesis [40].

#### GLRX5

Although the number of reported patients is still limited, two clearly distinct phenotypes, characterized either by isolated spasticity of the lower limbs or by isolated sideroblastic anemia, can be found. An erythroblastoid phenotype is expected, considering the abundant expression of GLRX5 in erythroid cells (CD71 +) and only minimal expression in other tissues [7]. However, in mice apart from erythroblasts high expression of GLRX5 was also demonstrated in the hippocampus and Purkinje cells of the cerebellum [7].

Three individuals with GLRX5 deficiency, caused by homozygous in frame deletion or out of frame insertion leading to premature stop codon in combination with the same in frame deletion, were identified in a cohort of patients with non-ketotic hyperglycinemia (NKH). In contrast to the classical presentation of NKH characterized by neonatal epileptic encephalopathy, these subjects developed symptoms much later and showed a milder disease course. One subject had only mild learning difficulties and two individuals had normal mental development, but one of them suffered from progressive deterioration of vision, in accordance with progressive optic nerve atrophy. Spasticity of the lower limbs occurred between the age of 2 and 7 years. These symptoms correlated with varying degrees of diffuse and progressive white matter alterations seen on brain MRI in two subjects, and only mild alteration of the upper spinal cord in another. All patients were still alive at the time of publication of the paper, i.e., aged between 7 and 11 years [41].

Two adults, both harboring missense mutations, were described with congenital sideroblastic anemia and hepatosplenomegaly, without signs of spasticity. Later, in the disease course, they developed diabetes mellitus type 1 [42, 43]. One patient had cirrhosis and hypogonadism [42].

Biochemical analysis in the subjects with the spastic phenotype showed increased glycine concentration in serum and cerebrospinal fluid (CSF). In accordance, the activity of glycine cleavage enzyme in liver tissue was low in all subjects. This was probably due to defective lipoylation of the H-protein moiety. Defective lipoylation of αKGDH and PDHC has already been demonstrated in cultured skin fibroblasts. Activity of OXPHOS complexes was not tested in all patients, but OXPHOS activities were not defective in cultured skin fibroblasts and skeletal muscle. Lactate was normal in these subjects [41].

In the probands with sideroblastic anemia, a significant amount (> 15%) of ring sideroblasts was detected in bone-marrow smears. Apart from erythroid iron accumulation, transferrin saturation and ferritin concentration were increased in serum. Further evidence of defective ISC biosynthesis was provided by decreased Fe/S incorporated in cytosolic aconitase and decreased catalytic activity of this enzyme [42, 43]. Activities of the mitochondrial aconitase and complex II were normal in lymphoblasts [41]. However, complex I activity and complex I expression were significantly decreased in cultured skin fibroblasts [7]. The cultured skin fibroblasts also showed increased iron accumulation, both in mitochondria and cytosol [7]. Interestingly, therapy with the iron chelator, deferoxamine, resulted in improvement of anemia in both patients [42, 43].

In HeLa cells depleted of GLRX5, a decreased activity of mitochondrial aconitase and xanthine oxidase was detected, confirming the essential role for cytosolic and mitochondrial ISC, both [4Fe–4S] and [2Fe–2S] clusters. These cells also showed increased (approximately doubled) total non-heme mitochondrial iron content and lower ferritin expression [7].

#### ABCB7

Deficient export of ISC or ISC intermediates results in iron accumulation in mitochondria. As synthesis per se is not affected, OXPHOS complexes are not deficient. Complementation studies in yeast showed the importance of ABCB7 protein for cytosolic ISC maturation. A defect in ABCB7 resulted in abolition of the activity of cytosolic aconitase, which functions as an iron regulatory protein (IRP), leading to increased transferrin receptor synthesis and subsequent increase in iron uptake. Anemia is explained by decreased ferritin and decreased erythroid 5-aminolevulinate synthase (ALAS2) synthesis.

All reported subjects with either hemizygous or heterozygous missense mutations presented with sideroblastic anemia in combination with ataxia. Although classically a non-progressive ataxia was reported, some adult patients suffered from regression of motor function. Early motor development varied from normal to delayed. Age of onset of the central nervous symptoms varied from early childhood to late adulthood. Ocular symptoms with nystagmus and/or small saccades were reported in three adults [44]. Cerebral imaging is normal or shows isolated cerebellar atrophy. Heterozygous females may suffer from hypochromic microcytic anemia, but do not present with neurological symptoms [45–48]. More recently, isolated cerebellar hypoplasia without sideroblastic anemia was reported in the affected individuals in one family. These individuals also harbored a deletion on chromosome X, affecting two other genes (*ATP7A* and *PGAM4*) that might have influenced the phenotype [49].

#### ALR

The ALR protein is encoded by the *GFER* gene. It is an oxidase essential for the mitochondrial disulfide relay system, which is extremely important for protein import into the mitochondrial intermembranary space [50]. ALR may be involved in export of ISC synthesized intermediates into the cytosol [37].

Affected subjects, all harboring missense mutations, have variable degrees of developmental delay, hypotonia and congenital cataract. In serum, lactate is increased.

In the first report, three subjects of consanguineous origin were described presenting with congenital cataracts, early onset progressive muscular hypotonia, sensorineural hearing loss, delay of motor skills and speech development [51]. In a second paper, an adult subject was described with infantile-onset adrenal insufficiency, cataract and subsequently poor feeding, irritability and hepatomegaly. Cerebral imaging revealed mildly increased signals bilaterally in the globus pallidus, which was resolved later on. By the age of 18 months the clinical situation stabilized and the child had only a slightly delayed development. At an early adult age, truncal hypotonia and muscle wasting were noticed, leading to respiratory insufficiency [52]. Very recently, two families, each with two affected siblings, were reported. Two siblings presented with regression at the age of 9 months associated with hypotonia evolving to severe developmental delay, dystonia, choreic movements and absent or minimal language development. Cerebral imaging showed moderate brain atrophy in one sibling. The other two subjects also presented with developmental delay associated with hypotonia and mild dystonic features. All reported subjects had congenital cataracts, and none had hearing loss [53].

When tested, serum lactate was found to be increased [51, 52]. Interestingly, OXPHOS testing in skeletal muscle revealed a combined OXPHOS deficiency involving complexes I, II, and IV in one subject [51] and deficiency of the OXPHOS complexes I, II, III, and IV in another [52]. A deficiency involving the complexes I, III, and IV was reported by Nambot et al. (2017) in two subjects. In another subject, only isolated complex IV deficiency was found in cultured skin fibroblasts [51]. Authors did not report anemia in the affected individuals, but three subjects were found to have low serum ferritin [51].

### Fe/S-cluster targeting and further maturation

After [2Fe–2S] clusters are released from the scaffold protein, they can immediately be incorporated into the apoproteins or the OXPHOS complexes I, II, and III. Incorporation needs the assistance of chaperone proteins. For further maturation to [4Fe–4S], assistance of a chaperone protein is also necessary, i.e., ISCA1 and ISCA2 as well as the folate binding protein IBA57 [54]. ISCA1 and ISCA2 are important for iron incorporation onto the [2Fe–2S]. NFU1 is a dedicated chaperone protein for [4Fe–4S] incorporation into complexes I and II as well as for LIAS (lipoic acid synthase), but not for mitochondrial aconitase. BOLA3 is another chaperone protein that has a similar role as NFU1. IND1 (encoded by *NUBPL*) which stands for the iron–sulfur protein required by NADH dehydrogenase, which was initially thought to be needed for incorporation of [4Fe–4S] clusters into complex I [55]. Its function was, however, reconsidered after studies with the *A. thaliana* ortholog Ind1, which showed that it functions as a translation factor necessary for expression of multiple complex I subunits [56]. Considering the uncertain nature of its action, we included a discussion on the clinical features associated with NUBPL dysfunction.

#### ISCA1

ISCA1 is one of the most recently reported defects in Fe/S-cluster biogenesis. Together with ISCA2 and IBA57 it acts at a late stage of the ISC biosynthesis pathway and is required for [4Fe–4S] cluster assembly. In two unrelated families with two affected children, ISCA1 deficiency caused by homozygous missense mutations was reported. The affected individuals had normal prenatal development, but in the young infantile period showed developmental delay, poor head control and signs of spasticity. Extensive cerebral and cerebellar abnormalities including pachygyria, enlarged lateral ventricles and abnormal cerebral and cerebellar white matter signals were seen on brain imaging. Seizures were apparent within the first months (2nd–5th) of life, and the children died between 11 months and 5 years. When documented, blood lactate was increased and a lactate peak was seen by cerebral MR spectroscopy [57]. Measurements of OXPHOS complex activities were not reported.

#### ISCA2

Although only 16 subjects with ISCA2 deficiency were reported until now, all caused by a missense mutation, an apparent uniform phenotype seems to segregate. Affected individuals presented a leukodystrophy characterized by diffuse white matter alterations seen on brain MRI extending into the corpus callosum and posterior limb of the capsula interna, and also alterations in the mesencephalon and cerebellar white matter. In some patients, abnormal cervical spinal cord signaling was also seen. The subjects became symptomatic after an uneventful pregnancy, between the age of three and 7 months. Presenting symptoms were loss of fixation with or without nystagmus and loss of acquired motor and social skills. Eventually all developed spasticity of the upper and lower limbs and optic nerve atrophy. All subjects were considered as having a degenerative condition and died a few months to 2 years after the onset of the initial symptoms [58, 59]. For some subjects, symptoms started after an inflammatory episode (infection or vaccine) or after mild head trauma [59]. CSF lactate and to a milder extent plasma lactate was increased. This was also the case for CSF and plasma glycine [59]. Analysis of OXPHOS complex activities in subject’s cultured skin fibroblasts revealed that complex I was severely impaired but complex II or III were normal. In HeLa cells depleted of ISCA2 using siRNA, a decreased expression of several ISC bearing proteins (mitochondrial aconitase, SDH, NDUFS3, NDUFA9, NDUFB4, NDUFA13, UQCRFS1) was found but no decrease of ferrochelatase. Activity measurements showed impaired activity of the complexes I and II and of mitochondrial aconitase. In accordance to its distal action in the ISC biosynthesis pathway, no deleterious effect on heme synthesis could be detected in ISCA2 depleted cells [58].

#### IBA57

Since the first publication in 2013 [60], four other papers were reported describing subjects with IBA57 deficiency, in total now 28 subjects. Three different phenotypes can be discerned with IBA57 deficiency, all with involvement of the central nervous system. The severity and type of lesions and the age of onset of symptoms are variable. Two phenotypes are associated with early fatal or debilitating outcomes [60–63], and one phenotype with a milder phenotype [64]. No genotype–phenotype correlations could be made. Most of the subjects harbored missense mutations. Insertions with frameshifts were also reported [63].

The first described subjects were siblings born from consanguineous parents presenting with intra-uterine growth retardation, polyhydramnion and microcephaly. At birth, they were hypotonic and presented with signs of encephalopathy. They had dysmorphic features, including retrognathia, high arched palate, widely spaced nipples, arthrogryposis of elbows, wrists, fingers and knees. Despite prompt adequate intensive support, the conditions deteriorated leading to early death. Cerebral MRI was abnormal with hypoplasia of the corpus callosum and medulla oblongata and bilateral frontoparietal polymicrogyria, and severely enlarged lateral ventricles [60].

Subjects with the mildest phenotype presented with spastic paraplegia, variably associated with optic nerve atrophy and peripheral neuropathy, abbreviated as SPOAN. Subjects suffered from slowly progressive gait impairment due to spastic paraparesis together with peripheral neuropathy and superficial sensory loss. Age of onset of gait impairment varied between 3 and 12 years. Central nervous system lesions were minor, as all affected subjects led an independent adult life, without cognitive impairment. Cerebral MRI in one subject showed, aside from bilateral optic nerve atrophy, scattered white matter alterations. Only one subject presented with mild cerebellar and cervical spinal cord atrophy [61].

In three reports, a third phenotype was described, all together in 15 subjects. The children presented with loss of motor and mental skills between 4 and 15 months of age. These findings correlated with extensive white matter alterations in cerebrum, cerebellum, mesencephalon and in the upper spinal cord. The corpus callosum and basal ganglia were not affected [61–63].

Increased lactate in serum and CSF and increased glycine were common biochemical features in most of the affected subjects. In all subjects, deficient activity and expression of complexes I and II and faulty lipoylation in all analysed tissues (lymphoblasts, cultured skin fibroblasts and skeletal muscle) were detected [60–63]. None of the described subjects had signs of anemia.

Similar as for ISCA2, HeLa cells depleted of IBA57 using siRNA, showed decreased expression of the ISC bearing subunits of complex I, complex II, and complex III but not of ferrochelatase.

#### NFU1

The role of NFU1 in human ISC biogenesis was deduced from the biochemical alterations detected in affected subjects. As complexes I and II and lipoylation were deficient, it was presumed that NFU1 acts as a chaperone dedicated to ISC incorporation in lipoic acid synthase, complex I and complex II. Expression studies demonstrated ubiquitous expression of the protein, with highest expression profiles in brain and heart, which is in parallel with mitochondrial content [65]. Missense [66–68] as well as splice site variants [68–70] were reported.

The first reported subjects presented with early onset encephalopathy and fatal outcome before the age of 1 month [66]. Ten other patients were reported with variable age of onset (between one and 9 months), and fatal outcome before the age of 15 months. This permitted a classification into three groups. In the first group, the affected subjects presented with failure to thrive, pulmonary hypertension, hypotonia and irritability. Cerebral MRI showed bilateral extensive white matter alterations. Recently, another paper confirmed the severe clinical picture in two children aged three and 4 months [71]. In a second group the affected individuals presented with pulmonary hypertension and regression of acquired skills after an intercurrent infection. A third group, with the mildest symptoms, showed only pulmonary hypertension and variable failure to thrive [70]. Ahting et al. (2015) described seven other subjects. All of them had central nervous system involvement with evolution into spastic tetraparesis and a declining clinical condition. Four of them suffered from pulmonary hypertension. One subject had dilated cardiomyopathy which became fatal at the age of 3 years [68]. Two individuals initially presented with early onset decline evolving to a stable spastic tetraparesis or paraparesis at adult age [69, 72].

The common feature for all reported subjects was an increased serum glycine and defective lipoylation resulting in decreased activity of complex II and PDHC (pyruvate dehydrogenase complex). Biochemical testing was not performed in the two most recently published cases [71].

#### BOLA3

Most reported subjects developed neurological symptoms at an early age, including seizures, leading to death before the age of 1 year, together with cardiomyopathy. Optic nerve atrophy was a variably occurring symptom [41, 66, 73]. When available, cerebral MRI showed various degrees of white matter alterations. Homozygous missense [41, 73] and homozygous duplication leading to a frameshift with premature a stop codon [66] have been reported.

Only one subject had a milder presentation with slowing of development starting at the age of 6 months. Subsequently, a slowly progressive spasticity and ataxia, as well as loss of language skills between 3 and 8 years of age were observed. Acute regression was seen at the age of 9 years with recurrent status epilepticus and worsening of spasticity. This subject finally died at the age of 11 years and was found to have only mild cerebral and cerebellar atrophy and no white matter changes [41].

Similar to NFU1 deficient subjects, increased lactate and glycine are a common feature of subjects with BOLA3 deficiency, together with defective lipoylation. Similar to NFU1 patients, the OXPHOS profile in cultured skin fibroblasts and skeletal muscle displayed a decreased activity of complexes I and II. When tested, PDHC was also deficient, but mitochondrial aconitase was normal [66, 73].

#### NUBPL

This protein is specifically dedicated to the incorporation of [4Fe–4S] clusters into complex I, leading to exclusive deficiency of complex I when NUBPL is deficient. Reported patients were all heterozygous for exonic missense mutations, deletions or insertions leading to a frameshift and premature stop codon. One intronic mutation was found to be located at intron 9–10 introducing a cryptic acceptor splice site leading to the introduction of an additional 72 bp, and finally resulting in a frameshift and nonsense mediated decay. This allele was found in heterozygous state with a complex genomic rearrangement detected in seven of the reported subjects [74–76].

The first reported subjects had isolated encephalopathy [74]. Another subject was described with progressive nystagmus, cerebellar ataxia and pyramidal tract signs in adult life. Cerebral MRI in the latter showed hyperintense lesions in the cerebellum, anterior mesencephalon and pyramidal tract [75].

A group of six patients was identified based on their cerebral MRI findings, showing extensive signal abnormalities in the cerebellar cortex, deep cerebral white matter and corpus callosum. Interestingly, cerebral white matter and corpus callosum abnormalities improved or even disappeared in the course of the disease, while cerebellar abnormalities became more extensive and abnormalities in the brainstem (basal pons and dorsal medulla oblongata) became visible. All subjects presented clinically with slow progression of motor function and ataxia, and in the majority of them with spasticity. Intellectual capacities varied from normal to severely impaired [76].

When reported, lactate was increased in plasma and CSF [76]. All described patients showed deficient complex I activity.

## Conclusion

Considering the strong evolutionary conservation of iron–sulfur clusters and its biosynthetic pathway throughout eukaryotes, it is not surprising that deficiencies in this synthesis pathway can cause severe impairment of cellular functioning and affect the viability of affected organisms. In this review we compiled the acquired knowledge on the clinical and genetic features of ISC deficiencies. Strikingly, a strict definable ‘ISC biosynthesis deficiency’ phenotype cannot be found. Most affected organs are the brain, resulting in developmental delay, epilepsy or regression, and skeletal muscle involvement leading to muscle weakness. Increased lactate and glycine in body fluids as well as anemia, eventually with the presence of sideroblasts, are possibly accompanying biochemical features associated with ISC deficiencies. This clinical and biochemical profile, except for increased glycine, is reminiscent of the clinical characteristics reported in the subjects with mitochondriopathies. The parallelism is not unexpected as three OXPHOS complexes harbor iron–sulfur clusters that function as electron carrier. Deficiencies in the cytosolic iron–sulfur cluster synthesis pathway have not been reported yet, and will probably result in clinical pictures different from those seen in CIA.
