# Correction to: Clinical and genetic aspects of defects in the mitochondrial iron–sulfur cluster synthesis pathway

**DOI:** 10.1007/s00775-018-1564-6

**Published:** 2018-05-31

**Authors:** A. V. Vanlander, R. Van Coster

**Affiliations:** Division of Pediatric Neurology and Metabolism, Department of Pediatrics, Ghent University Hospital, Prinses Elisabeth ziekenhuis, 3K12D, secretariaat kinderneurologie, Corneel Heymanslaan 10, Ghent, 9000 Belgium

## Correction to: JBIC Journal of Biological Inorganic Chemistry 10.1007/s00775-018-1550-z

The article “Clinical and genetic aspects of defects in the mitochondrial iron–sulfur cluster synthesis pathway”, written by A. V. Vanlander, R. Van Coster was originally published electronically on the publisher’s internet portal (currently SpringerLink) without open access.

The copyright of the article changed on 19, May to © The Author(s) 2018 and the article is forthwith distributed under the terms of the Creative Commons Attribution 4.0 International License (http://creativecommons.org/licenses/by/4.0/), which permits use, duplication, adaptation, distribution and reproduction in any medium or format, as long as you give appropriate credit to the original author(s) and the source, provide a link to the Creative Commons license and indicate if changes were made.

The original article has been corrected.

